# Cross-cultural adaptation and psychometric evaluation of the Slovenian version of the nurse professional competence scale

**DOI:** 10.1186/s12912-021-00664-6

**Published:** 2021-08-11

**Authors:** Mirko Prosen, Andreja Kvas, Sandra Bošković, Sabina Ličen

**Affiliations:** 1 Department of Nursing, Faculty of Health Sciences, University of Primorska, Polje 42, 6310 Izola, Slovenia; 2 Department of Nursing, Faculty of Health Sciences, University of Ljubljana, Zdravstvena pot 5, 1000 Ljubljana, Slovenia; 3 Department of Nursing, Faculty of Health Studies, University of Rijeka, Viktora Cara Emina 5, 51000 Rijeka, Croatia

**Keywords:** Instrument Validation, Professional Competence, Care, Quality, Safety

## Abstract

**Background:**

The competency-based approach to the assessment of nursing practice has been adopted as a key policy in the developed world. The continual self-assessment of competence gives nurses the opportunity to reflect on their competencies and has a significant impact on the quality of nursing practice and patient safety. The study was designed to describe the process of cross-cultural adaptation and to assess the psychometric properties of the Slovenian version of a short form of the Nurse Professional Competence scale (NPC-SF) and to evaluate the efficacy of this instrument in a sample of registered nurses.

**Methods:**

A cross-sectional and validation study was conducted in 425 registered nurses to test the psychometric properties of the Slovenian version of a short form of the scale and to evaluate nurses’ professional competence. A multilevel approach was used: Translation, back-translation, language validity, face and content validity, construct validity, and reliability of the Slovenian version of the scale were analysed respectively. Participants completed an online survey, with the data being collected between April and July 2020.

**Results:**

Factor analysis showed that the Slovenian version of the scale could be used in four dimensions explained with 65 % of the variance. Cronbach’s α was 0.972. The four-factor model fit the data (RMSEA = 0.083, CFI = 0.731). Self-reported competence was high and rated higher by nurses employed at the tertiary level of healthcare, followed by nurses employed at the secondary and primary, and from social care institutions. Nurses with more years of experience assessed their competence higher.

**Conclusions:**

The NPC-SF helps understand and identify nurses’ self-reported core competencies in clinical settings, thereby providing an important predictor of the professional development of nursing. The Slovenian version of the scale demonstrated acceptable psychometric properties and may be used in research and clinical practice to evaluate nurses’ professional competence.

## Introduction

Parallel to cuts in healthcare spending and insufficient time to establish strong relationships with patients, nurses are ever more concerned about patient safety, the quality of patient care, and their own safety and protection [1]. This has led to a bigger emphasis on nurses’ professional qualifications since nursing care, that is high in quality and safe, depends on the professional competency nurses hold [2, 3]. Professional competencies are not only recommended from an academic perspective but are also part of the legal requirements for professional registration and practice standards. The reason competencies are so vital [4] is that one’s professional performance depends heavily on one’s knowledge and skills [5] within an organisational environment governed by responsibilities and standards [4]. Nobahar [6] believes that the possession of a suitable set of skills leads to greater quality patient care and increased patient satisfaction with nurses’ work. This in turn helps promote nursing as a profession and influences nurses to improve their educational attainment and clinical practice.

## Background

Nurses should acquire and develop appropriate competencies while studying and later through their professional practice [7]. On the European level, the competencies needed for registered nurses are emphasised in various nursing standards: e.g., the updated Directive 2013/55/EU (Official Journal of the European Union) and the Tuning Framework in Europe [8]. The field of nursing education is also led by international associations such as the International Council of Nurses (ICN), the World Health Organization for Europe (WHO for Europe), the European Federation of Nurses (EFN) and the European Federation of Nursing Educators (FINE), etc. [9]. In Slovenia, two mechanisms help to ensure nursing competences when starting practice: accreditation of nursing education programs and professional licensing [10]. Besides, some authors state [2, 6] that the clinical environment in which an individual gains experience by working, observing, and translating scientific knowledge into practice is also crucial for the development of competencies in nursing. The level of competence acquired strongly influences the provision of nursing care. However, despite solid evidence that high professional qualifications lead to greater patient safety [11], not enough nurses have accomplished higher education levels (i.e., specialisation, master’s, or doctoral degrees) to meet and satisfy the patient-safety requirements [12].

Nursing as a profession in Slovenia, despite the progress it has made over the past 30 years, still faces some obstacles to professionalization, and it is only since 2017 that nurses have been able to obtain a doctorate in nursing. Moreover, after years of attempts, in 2019 Slovenia finally obtained a document outlining nurse competencies according to the level of educational attainment [13]. The definition of competence used in this study is the same as in the Slovenian document “Professional competencies and activities of nursing professionals” [13], where the term competence refers to the ability to use knowledge and other skills as required to perform a given task successfully and efficiently, for the performance of work, to achieve goals or the fulfilment of a specific role in the work process. In preparing the document, the international guidelines (ICN, EFN) for describing the level of competence in nursing were taken into account, as this was the most appropriate given the history of nursing education in Slovenia and the existing systematization of work positions in nursing in clinical practice [13–15].

In this context, nurses’ professional skill set might also affect their attitudes to their work, including their organisational commitment and professional affiliation. Additionally, nurses with a higher level of clinical competence have been shown to reduce patient mortality rates within hospital settings [11, 16, 17]. In clinical practice, there are several approaches that could indicate high nurse expertise, such as “nursing-sensitive patient outcomes, medication administration errors, patient falls, nosocomial infections, patient complaints, pressure ulcers, cardiopulmonary arrests, standardized by patient days or doses of medication” [17] or self-perception methods [18]. Continuous evaluation of even these competency standards is therefore high on the agenda of strategic health planners [19]. A comparison with other studies showed that nursing competencies are idiosyncratic nationally, mainly due to differences in traditions of nursing culture, nursing process, nursing education or the national legal competency framework [9]. Measuring nursing competences must be valid, reliable, accurate, and responsive (i.e., able to reflect a valid change in a construct of interest) [20].

However, when it comes to professional nursing competencies and the rating scales we use to assess them, we often rely on foreign literature and findings that are often incompatible with the context of nursing practice in Slovenia. There are several scales to measure the attainment of professional competence, but considering the context and competencies of nurses in Slovenia, the Nurse Professional Competence Scale (NPC) [21] seems the most appropriate.

This study aimed to describe the process of cross-cultural adaptation and psychometric validity of the Slovenian version of a short form of the Nurse Professional Competence scale (NPC-SF) and to evaluate the efficacy of this instrument in a sample of registered nurses.

## Methods

### Study Design

A cross-sectional and validation study was designed to evaluate a short form of the Nurse Professional Competence Scale. While translating and adapting the NPC-SF scale, the accepted translation procedures recommended by the original scale’s authors [22] were followed together with the WHO’s process of instrument translation and adaptation [23].

### Participants and settings

A convenience sample of 425 registered nurses was recruited to participate in the study based on a snowballing method, of which 75 (17.6 %) were male and 350 (82.4 %) were female. The calculated sample size based on 95 % confidence level with 5 % margin of error was 367. All participants were from clinical practice, i.e. primary, secondary and tertiary levels of healthcare and social care institutions. The inclusion criteria were: To be a registered nurse currently working in a clinical setting in one of the three levels of healthcare or social care institutions. The exclusion criteria were: To be a registered nurse who is not currently working or has been in service for less than one year. Their average age was 34.3 years (SD = ± 10.9) and average number of active years working as a registered nurse was 14.4 (SD = ± 11.6). The study was conducted between April and July 2020. Registered nurses received an e-mail invitation explaining the study’s purpose, assuring that participation was voluntary, and there would be no consequences for not participating in the study.

### Instrument

The NPC scale was originally developed on the basis of the formal skills required of registered nurses, which are in turn influenced by the World Health Organisation (WHO) – European Strategy for Nursing and Midwifery in order to improve and standardise educational programmes for nurses and midwives. The initial NPC scale consists of 88 items and covers 8 areas of competence: nursing care, value-based nursing care, medical/technical care, teaching/learning and support, documentation and information technology, legislation in nursing and safety planning, leadership in nursing and the development of nursing care, and education and supervision of staff/students, with Cronbach’s alpha values ranging from 0.75 to 0.94 for individual competence areas [18]. Later, in 2017, a short form of the scale (NPC-SF) was developed to facilitate its further use in evaluations and research regarding the self-reported professional competence of nursing students and registered nurses [21]. The NPC-SF consists of 35-item in six factors, labelled: (i) Nursing Care; (ii) Value-based Nursing Care; (iii) Medical and Technical Care; (iv) Care Pedagogics; (v) Documentation and Administration of Nursing Care; and (vi) Development, Leadership and Organisation of Nursing Care. The response format for rating the scale is based on seven answer alternatives for each item, from 1 = to a very small degree to 7 = to a very high degree.

### Data collection

Registered nurses agreed to participate in the study by clicking on an embedded link and completing an electronic survey. They had the opportunity to complete the survey within 4 months, from April to July 2020. The average time to complete the survey was 8 min. The database contained the respondents’ answers without their surnames, first names or e-mail addresses, thereby guaranteeing anonymity. Survey reminders followed every 2 weeks after the invitation had been distributed. The data provided by the respondents by answering the questions was collected in a database on the 1 ka.si web server and, when the survey was completed, all data were simultaneously transferred and exported to SPSS for statistical analysis purposes.

### Translation and adaptation process

#### Translation of the original scale

The process started by translating the original instrument. The scale was first translated from English into the Slovenian language. Experts from the field of nursing and quantitative research methods were involved in preparing the first Slovenian version of the scale, given that the translation of individual items should not be literal but semantic to ensure conceptual and linguistic equivalence. A back-translation followed, with the Slovenian version of the questionnaire being translated back into English. At this point, the scale was handed over to a bilingual translator of English origin. After back-translating the questionnaire, the bilingual translator and research group members met again to evaluate the translated and back-translated versions and to assess the scale’s semantic and conceptual aspects.

#### Face validity assessment

The face validity of the questionnaire was then evaluated by asking 10 registered nurses from the clinical environment to assess the items and critically review them with regard to their understanding. All of the respondents’ comments were further analysed.

#### Content validity assessment

The content validity index (CVI) of the scale was assessed according to the approach proposed by Lindell and Brandt [24]. The translated NPC-SF scale was distributed to 8 faculty members (i.e., professionals in nursing science and experts in the topic under study). According to Polit et al. [25] the acceptable CVI values should be at least 0.75. The experts were asked to determine the relevance, and clarity of each item on a 4-point Likert scale: completely relevant (4), somewhat relevant (3), needs serious revision (2), and irrelevant (1) and 3-point Likert scale was used for the clarity: 1 = not clear, 2 = item needs some revision; and 3 = very clear. The CVI of each item was calculated by dividing the number of experts who rated that item 3 or 4 by the total number of experts [26].

#### Construct validity assessment

The scale was then evaluated for construct validity using confirmatory factor analysis (CFA). The Kaiser-Meier-Olkin (KMO) measure was used to test the sample’s adequacy. A value of > 0.6 for KMO is considered good (26), while factor loadings of > 0.71 are considered excellent. Within the structural equation modelling (SEM), Goodness-of-fit indexes, the Chi-square test (χ2), the Root mean square error of approximation (RMSEA) and the Comparative fit index (CFI) were used. A χ2 test > 0.05 is desirable, although when a large sample is used the χ2 test is often significant and researchers therefore recommend using RMSEA (< 0.08 has been reported as acceptable and values < 0.06 as good) [27].

#### Reliability assessment

Reliability measured as internal consistency was evaluated using Cronbach’s alpha. Values of $\ge$ 0.70 were assessed as sufficient [28].

### Data analysis

The translated 35-item scale was evaluated with respect to several psychometric properties: face validity, content validity, construct validity and reliability. For statistical analysis purposes, the data were exported to IBM SPSS, version 26.0, and further to IBM SPSS Amos, version 26.0 (SPSS Inc., Chicago, IL USA). The following statistical analyses were performed to determine the scale’s psychometric properties: Cronbach’s alpha coefficient to establish internal consistency, confirmatory factor analysis and Pearson’s correlation coefficient. Descriptive statistics were further used to describe and summarize. The normality of the data was tested by Kolmogorov-Smirnov test. Since the data were not normally distributed (p < 0.0001), both the nonparametric Kruskal Wallis H-test and the Mann-Whitney U-test were used to establish statistically significant differences between groups. Additionally, linear regression was used to test relationships between categorical variables. A p-value ≤ 0.05 was considered significant.

### Ethical considerations

The principles established in the Declaration of Helsinki [29] were followed. The study was approved by the Ethical Committee of the XXX (No. 4/10/18; 29 October 2018). All data were treated confidentially. The original NPC-SF scale and permission to use it for research purposes were granted by the original scale’s authors, while permission was also obtained from the participating healthcare institutions that participated in the online dissemination of the adapted scale.

## Results

The study respondents were 425 registered nurses. While there is no general agreement on the appropriate sample size for factor analysis, it is noted that the minimum number is at least 100, or 3 to 20 times the number of variables [30].

The results are presented below by validation phase.

The translated scale was further prepared for the face validity phase. Here, the scale was evaluated by 10 registered nurses from a clinical environment. The respondents found that all 35 questionnaire statements were written clearly, concisely, and comprehensibly. After some small text corrections to ensure terminological adequacy and address spelling mistakes, the final version of the Slovenian scale was drafted. The scale is named the Slovenian version of the Nurse Professional Competence Scale (Sl-NPC-SF).

Subsequently, the NPC-SF scale was reviewed for content validity by a total of 8 scientific experts in the field of nursing. A CVI of 0.75 or higher was considered acceptable. The CVI of the items ranged from 0.88 to 1, and the scale CVI was 0.98. Based on the results, none of the items were excluded from the further validation process.

In the next phase, CFA was performed. The application of factorial analysis was considered appropriate with the Kaiser-Meyer-Olkin (KMO) index = 0.96 and Bartlett’s test of sphericity being significant (χ2 = 9014.86, df = 595, p < 0.000). Exploratory factor analyses resulted in four factors explaining 65 % of the variance. The rotated component matrix (Table 1) showed the factor loads were greater than 0.4 ($\ge$0.35 is considered acceptable) [31].

**Table 1:** Factorial structure 4-factors, 35 items

***Do you think you have the ability to ....***		Factor loadings	Mean score (SD)	I-CVI (R, C)
**Factor 1**	Supervise and train co-workers/staff?	0.775	5.21 (1.439)	1
	Teach, supervise and assess students?	0.760	5.37 (1.377)	1.0
	Inform and educate patients and next of kin in a group, taking into account time, form and content?	0.721	5.40 (1.177)	1.0
	Inform and educate patients and next of kin individually, taking into account time, form and content?	0.691	5.59 (1.106)	1.0
	Make sure that the patient and next of kin understand the information provided?	0.664	5.76 (0.950)	1.0
	Implement new knowledge and thus promote nursing care in accordance with science and evidence-based practice?	0.633	5.34 (1.159)	0.94
	Plan, consult, inform and cooperate with other actors in the chain of care?	0.592	5.57 (1.070)	1.0
	Systematically lead, prioritise, delegate and coordinate nursing care within the team, based on the patient’s needs and the different competencies of co-workers/staff?	0.574	5.40 (1.201)	1.0
	Make use of relevant patient records?	0.536	5.76 (0.947)	1.0
	Carry out documentation according to current legislation?	0.504	5.34 (1.130)	1.0
	In dialogue motivate the patient to comply with treatments?	0.489	5.57 (1.021)	1.0
	Provide support and guidance to enable optimal participation in care and treatment, in dialogue with the patient and next of kin?	0.484	5.66 (1.126)	0.88
	Use information and communication technology (ICT) to support nursing care?	0.459	5.32 (1.245)	1.0
**Factor 2**	Show concern and respect for the patient’s autonomy, integrity and dignity?	0.786	6.24 (0.917)	1.0
	Communicate with patients, next of kin and staff respectfully, sensitively and empathetically?	0.724	6.22 (0.956)	1.0
	Show openness to and respect for different values and faiths?	0.709	6.13 (0.911)	1.0
	Utilise the knowledge and experience of the team and others, and through team collaboration contribute to a holistic view of the patient?	0.636	6.09 (0.943)	1.0
	Handle sensitive information correctly and carefully?	0.539	6.09 (0.966)	1.0
	Continuously engage in your own personal and professional competence development?	0.538	5.73 (1.033)	1.0
	Utilise the knowledge and experience of the patient and/or their next of kin?	0.516	5.82 (1.012)	1.0
**Factor 3**	Manage drugs adequately, applying knowledge in pharmacology?	0.690	5.17 (1.329)	1.0
	Question unclear instructions/prescriptions?	0.622	5.36 (1.219)	0.94
	Independently administer prescriptions?	0.607	3.62 (1.567)	0.94
	In case of a serious incident within or outside the care institution, apply emergency medical principles?	0.590	5.47 (1.183)	1.0
	Handle medical products on the basis of existing regulations and safety routines?	0.588	5.66 (1.132)	1.0
	Act adequately in case of unprofessional conduct by staff?	0.508	5.31 (1.240)	1.0
	Pay attention to work-related risks and actively prevent these?	0.499	5.59 (1.003)	0.94
	Comply with existing regulations as well as guidelines and procedures?	0.494	5.62 (1.018)	1.0
	Display judgement, knowledge and thoroughness when informing and providing for the patient’s security and wellbeing during examinations and treatments?	0.417	5.66 (1.056)	1.0
**Factor 4**	Cater for the patient’s needs regarding specific, physical nursing care?	0.767	5.70 (1.041)	1.0
	Cater for the patient’s needs regarding basic, physical nursing care?	0.740	6.04 (0.972)	1.0
	Document the patient’s physical condition?	0.726	5.80 (1.075)	1.0
	Independently apply the following stages in the nursing process: observation and assessment (nursing anamnesis, status and nursing goals)?	0.667	5.39 (1.186)	1.0
	Document the patient’s psychological condition?	.0576	5.22 (1.214)	1.0
	Follow up the patient’s condition after examinations and treatments?	0.493	5.63 (1.121)	1.0

Accumulated total explained variance = 65%. Bartlett’s Test of Sphericity: *χ2* = 9014.86, p < 0.0001; Kaiser- Meyer- Olkin value = 0.96; SD – Standard Deviation; F1 – Development leadership and organisation of nursing care; F2 – Value-based nursing care; F3 – Documentation and administration of nursing care; F4 – Nursing care; Rating the scale is based on seven answer alternatives, from 1 = to a very small degree to 7 = to a very high degree; I-CVI (R, C) – Items Content Validity Index (Relevancy, Clarity)

**Fig. 1:**
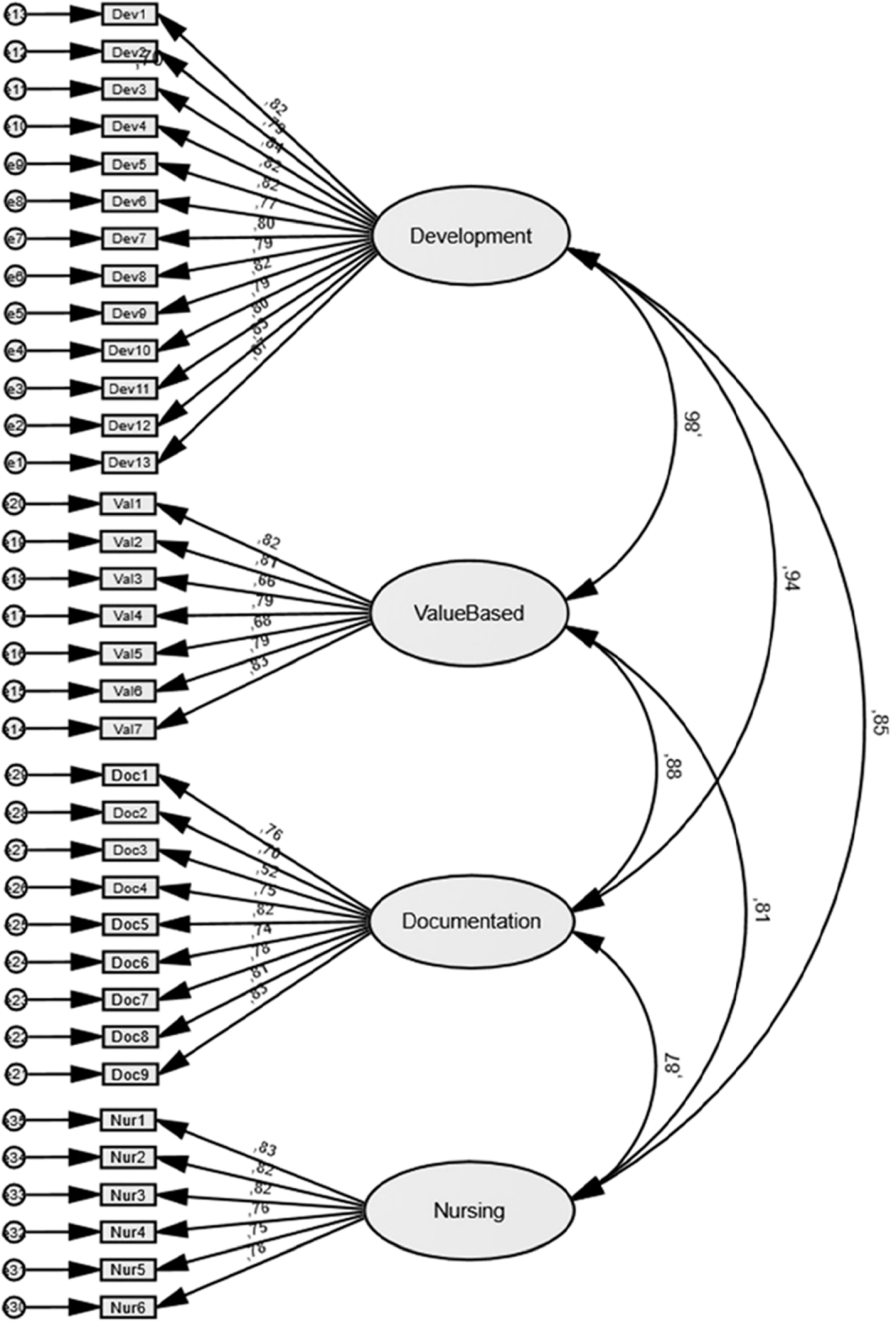
Flow chart presenting the modelling construct of the 35-item Sl-NPC-SF scale

Each factor was examined for content within the items and thereafter the four factors were named: Factor 1: Development leadership and organisation of nursing care (13 items); Factor 2: Value-based nursing care (7 items); Factor 3: Documentation and administration of nursing care (9 items); and Factor 4: Nursing care (6 items). The results also showed that the fit estimates that the four-factor measurement model’s compliance with the Sl-NPC-SF scale is appropriate. The χ2 test for the four factors was 2165.36 (df = 554, p < 0.000). A significant χ2 is common in a large sample. The RMSEA was 0.083 (the cut-off point for a good fit is < 0.08) and the 90 % confidence interval was 0.079 to 0.086. A more encouraging result is that CFI was 0.731, indicating a good fit while reaching the recommended value of > 0.90 [27]. Based on standardised values, the results reveal a very high weighting of the indicators for the latent variables (from 0.52 to 0.94) (Fig. 1).

The inter-correlation of the four factors was tested, with the findings showing a positive and strong correlation between the variables, ranging from 0.808 to 0.945 (M = 0.863). All correlations were statistically significant *p* < 0.001 (Table 2).

**Table. 2:** Inter-factor correlation matrix of the Sl-NPC-SF

Factors	F1	F2	F3	F4
(F1) Development leadership and organisation of nursing care		0.861^**^	0.945^**^	0.846^**^
(F2) Value-based nursing care	0.861^**^		0.875^**^	0.808^**^
(F3) Documentation and administration of nursing care	0.945^**^	0.875^**^		0.866^**^
(F4) Nursing care	0.846^**^	0.808^**^	0.866^**^	

Correlation is significant at the 0.05^*^ and 0.01^**^ levels

The assessment of the Sl-NPC-SF Scale identified by the registered nurses are (M = 5.66, SD = 0.872 (95 % CI 5.46, 5.65), *p* = 0.000). A reliability test was conducted using consistency and stability tests on the scale’s four factors. The results indicated excellent reliability with regard to all four factors and for the entire Sl-NPC-SF scale (Table 3).

**Table. 3:** Scale summary data and psychometric properties (*N* = 425)

Scales / clinical settings	n	Cronbach α	M	SD	95 % CI	P value
Development leadership and organisation of nursing care (F1)	13	0.950	5.538	0.964	5.376–5.581	< 0.001
Value-based nursing care (F2)	7	0.892	6.142	0.786	5.965–6.128	< 0.001
Documentation and administration of nursing care (F3)	9	0.904	5.333	0.933	5.163–5.361	< 0.001
Nursing care (F4)	6	0.902	5.666	0.938	5.523–5.718	< 0.001
Total Sl-NPC-SF scale	35	0.972	5.657	0.872	5.464–5.645	< 0.001

*M* Median; *SD* Standard Deviation; *95 % CI* 95 % Confidence Interval; The Guttman split-half coefficient of the Sl-NPC-SF scale was 0.938; Spearman–Brown coefficient = 0.939.

To examine the relationship between participants in terms of their gender and level of healthcare in relation to the Sl-NPC-SF scale, the Mann-Whitney U test, the Kruskal-Wallis H test, and linear regression were performed (Table 4).

**Table. 4:** Characteristics of the sample population concerning the Sl-NPC-SF scale and the four subscales (*N* = 425)

**Variable**	**Factor 1**	**Factor 2**	**Factor 3**	**Factor 4**	**NPC-SF**
	M(SD)				
	Test value* / p*				
Gender					
Male	5.769(1.007)	6.142(1.016)	5.666(0.996)	5.666(1.083)	5.857(0.973)
Female	5.666(0.889) (U)3342.00 / *0.153*	6.142(0.701) (U)4207.00 /*0.722*	5.444(0.851) (U)2958.50 / *0.016*	5.833(0.831) (U)4256.00 / *0.811*	5.742(0.757) (U)3739.00 / 0.142
Health facilities					
Primary Health Care	5.692(0.842)	6.285(0.764)	5.333(0.817)	5.666(0.906)	5.685(0.721)
Secondary Health Care	5.538(0.903)	6.142(0.764)	5.444(0.851)	5.833(0.767)	5.714(0.775)
Tertiary Health Care	5.846(1.013)	6.166(0.880)	5.666(0.979)	6.000(0.916)	5.866(0.880)
Social Welfare Institution	5.384(0.668) (H)1.916 / *0.590*	6.000(0.757) (H)2.395 / *0.495*	5.555(0.731) (H)4.649 / *0.199*	5.833(0.668) (H)7.638 / *0.054*	5.628(0.645) (H)3.290 / *0.349*

F1 – Development leadership and organisation of nursing care; F2 – Value-based nursing care; F3 – Documentation and administration of nursing care; F4 – Nursing care; Median (M); Standard deviation (SD); U value - Mann-Whitney U test; χ2 value - Kruskal Wallis H test; χ2 value - Pearson chi-square test; p value - statistical significance

Although the results show some differences between the groups, the results are not statistically significant (p > 0.05) in relation to gender and the level of healthcare they are currently working, with the exception of the subscale »documentation and administration of nursing care«, in which male nurses rated their professional competence higher than their female colleagues (p < 0.05).

In addition, a linear regression analysis was performed to predict the Sl-NPC-SF assessment score based on participants’ length of professional experience. Here, a significant regression equation was found for the total Sl-NPC-SF scale (F(1, 240) = 26.309, *p* < 0.000), with R^2^ of 0.099, in the development leadership and organisation of nursing care subscale (F(1, 229) = 23.761, p < 0.000), with R^2^ of 0.094, in the value-based nursing care subscale (F(1, 240) = 7.381, *p* < 0.007), with R^2^ of 0.030, in documentation and administration of nursing care subscale (F(1, 229) = 32.489, p < 0.000), with R^2^ of 0.124, and in the nursing care subscale (F(1, 240) = 71.269, p < 0.000), with R^2^ of 0.067, indicating that as participants’ length of service increased, nurses rated their professional competence higher. However, the influence of length of service on the level of competence assessment in the total scale and in all subscales is small.

## Discussion

The Sl-NPC-SF enables nurses to self-report or assess their professional competence. The aim of this study was to describe the process of cross-cultural adaptation, to validate the short form of the NPC scale and to assess nurses’ professional competence in Slovenia.

The results for the Sl-NPC-SF show that its psychometric properties are adequate in terms of face and content validity, internal consistency, temporary stability, reliability, and construct validity, despite the factor structure having changed from six to four factors. The model is now focused on the development, leadership and organisation of nursing care, value-based nursing care, documentation and administration of nursing care and nursing care and reflects the current practice of nursing and nurses’ role in Slovenia. The four-factor model explained 65 % of total variance compared to the original version whose six-factor solution explained 53.6 % of total variance [21]. These four factors correspond well with the competency description used in the Slovenian document [13], which in turn is structured according to the core nursing competencies described by EFN Competency Framework: culture, ethics and values, health promotion and prevention, guidance and teaching, decision-making, communication and teamwork, research, development and leadership, and nursing care [32]. In the 35-item Sl-NPC-SF scale, the four competencies as well as two missing factors from the original version, medical-technical care and care pedagogics are visible and captured when using the scale. Further, the Sl-NPC-SF showed excellent internal consistency [33] for both the individual components and the entire instrument. The results also show that nurses rate their self-reported competence highly and differently depending on the level of healthcare at which they are employed at. Additionally, the results show a statistically significant impact of length of service on nurses’ competency ratings, as nurses with longer work experience rated their competencies higher. Previous research has shown that nurses’ self-assessment of competencies differs in relation to the length of work experience and that nurses with more than 6 years of work experience rated their competencies higher [34].

The Sl-NPC-SF had a high response rate, increasing the study’s reliability [33]. We may assume similarly as the instrument’s authors did when using the short version [21] that the response rate increases as the instrument becomes shorter. The instrument’s shortness is another advantage because it allows the researcher to combine the NPC-SF with other instruments, e.g., objective measures to obtain more specific information about nursing competence [12, 21].

The concept of nursing competence remains a central tenant of the scope of nursing practice, with the underlaying premise that patients are entitled to receive high-quality care from competent nurses [35] who meet the regulatory requirements of the nursing profession and standards of practice [12, 36]. The outcomes of this association between quality of care and nurse competence can be measured by nursing-sensitive patient outcomes and, as nursing skills increase, adverse occurrence rates should decrease [36]. European Directive 2005/36/ EC states that general nursing education should last at least 3 years or 4.600 h, which includes both theoretical and clinical components of education. Within the hours devoted to clinical education, nursing students gain experience and develop skills by working in clinical settings [9]. Today’s nursing students are expected to have appropriate competencies upon graduation, which means that professional competencies are also an important outcome of nursing education and appropriate assessment of these competencies can also be helpful in promoting the quality of nursing education [10, 37].

Evaluating the professional competence of nurses is extremely important in settings where the nursing profession is still trying to achieve its professional status and faces several barriers in its professionalization. To analyse the complex nature of competencies, competence assessment scales must be valid, reliable, and responsive [36]. Slovenia lacks validated assessment tools for measuring the professional competence of nursing students and registered nurses. The original version of the NPC-SF consists of 35 items grouped within a 6-factor structure (Nursing care, Value-based nursing care, medical and technical care, care pedagogics, documentation and administration of nursing care and development, leadership, and organisation of nursing care) and was developed to explore professional competence in nursing [21]. We chose this instrument since the NPC-SF’s content and six-factor structure corresponds well with the competencies described by the Nurses and Midwives Association of Slovenia which are aligned with international policy documents establishing the contemporary scope of nursing practice [13]. The instrument has also proven its worth and applicability in international contexts, providing an additional reason for choosing it for the research [12].

Professional nursing competence is one of the essential quality-of-care standards, and its assessment can lead to the identification of areas of nursing still requiring improvement. In addition, assessment of professional competence is the most important predictor of nurses’ professional development to provide safe, effective, and professional care to patients. Assessment of professional nursing competence can lead to the identification of areas of nursing that still need further improvement. The findings suggest that leadership and management competencies are among the most important competencies for nurses in the current nursing context and should be further explored, especially in certain specific areas that are often neglected, such as risk management, research, quality, and finance. Besides, the Sl-NPC-SF could also be used in further research at the national level or in collaborative research with educational providers, employers, and professional organisations as there are still many challenges that remain unclear within the concept of nursing competency.

## Limitation

This study has some limitations. One limitation is that, notwithstanding the high response rate, the results cannot be generalised for the Slovenian context and more research is needed to do so. Accordingly, another limitation to be considered is the sampling approach that did not focus sufficiently on representativeness. Instead of a convenience sample, future research should rely on a stratified sampling technique to allow gaps in the self-reported competence of student nurses and nurses to be observed.

Moreover, as has been suggested [21], a seven-point response scale fits the NPC-SF best since it increases its reliability, with the same having already been applied in the Sl-NPC-SF; still, the Sl-NPC-SF was not then tested with other response alternatives to be able to draw valid conclusions. Future research on the Sl-NPC-SF should therefore include broader analysis and further testing of the factor structure to check changes in the reduced-form model in comparison to the original version.

## Conclusions

The study results show the Sl-NPC-SF instrument is a valid and reliable tool offering several advantages, including that it is easy to apply while investigating the self-reported competence of nurses. The Sl-NPC-SF was tested and analysed in a rigorous way, adding credibility to the instrument. Despite further refinement, the Sl-NPC-SF is leading to new evidence concerning the validity and reliability of existing NPC scales.

### Relevance to Clinical Practice

Professional nursing competence is one of the essential quality-of-care standards, and its assessment can lead to the identification of areas of nursing still requiring improvement. Moreover, assessment of professional competence is the most important predictor of nurses’ professional development, and the use of the Sl-NPC-SF scale could change assessment policies by providing regular self-assessment of nurses’ professional competence considering specific cultural context of nursing profession. In addition, the Sl-NPC-SF could also be used in further research at a national level or in collaborative research with educational providers, employers, and professional bodies as there are still many challenges that remain unclear within the concept of nursing competence.

## Data Availability

The datasets generated and/or analysed during the current study are not publicly available due institutional data sharing clause but are available from the corresponding author on reasonable request.
